# Self-blame in major depression: a randomised pilot trial comparing fMRI neurofeedback with self-guided psychological strategies

**DOI:** 10.1017/S0033291721004797

**Published:** 2023-05

**Authors:** Tanja Jaeckle, Steven C. R. Williams, Gareth J. Barker, Rodrigo Basilio, Ewan Carr, Kimberley Goldsmith, Alessandro Colasanti, Vincent Giampietro, Anthony Cleare, Allan H. Young, Jorge Moll, Roland Zahn

**Affiliations:** 1 Department of Psychological Medicine, Centre for Affective Disorders, London, UK; 2 Department of Neuroimaging, Institute of Psychiatry, Psychology & Neuroscience, King's College London, London, UK; 3 Cognitive and Behavioral Neuroscience Unit and Neuroinformatics Workgroup, D'Or Institute for Research and Education (IDOR), Rio de Janeiro, Brazil; 4 Department of Biostatistics, Institute of Psychiatry, Psychology & Neuroscience, King's College London, London, UK; 5 South London and Maudsley NHS Foundation Trust, Bethlem Royal Hospital, Monks Orchard Road, Beckenham, Kent, BR3 3BX, UK

**Keywords:** Anger, anterior temporal lobe, Brodmann Area 25, guilt, major depressive disorder, neurofeedback, psychotherapy, real-time fMRI, social cognition, subgenual cingulate cortex

## Abstract

**Background:**

Overgeneralised self-blame and worthlessness are key symptoms of major depressive disorder (MDD) and have previously been associated with self-blame-selective changes in connectivity between right superior anterior temporal lobe (rSATL) and subgenual frontal cortices. Another study showed that remitted MDD patients were able to modulate this neural signature using functional magnetic resonance imaging (fMRI) neurofeedback training, thereby increasing their self-esteem. The feasibility and potential of using this approach in symptomatic MDD were unknown.

**Method:**

This single-blind pre-registered randomised controlled pilot trial probed a novel self-guided psychological intervention with and without additional rSATL-posterior subgenual cortex (BA25) fMRI neurofeedback, targeting self-blaming emotions in people with insufficiently recovered MDD and early treatment-resistance (*n* = 43, *n* = 35 completers). Participants completed three weekly self-guided sessions to rebalance self-blaming biases.

**Results:**

As predicted, neurofeedback led to a training-induced reduction in rSATL-BA25 connectivity for self-blame *v.* other-blame. Both interventions were safe and resulted in a 46% reduction on the Beck Depression Inventory-II, our primary outcome, with no group differences. Secondary analyses, however, revealed that patients without DSM-5-defined anxious distress showed a superior response to neurofeedback compared with the psychological intervention, and the opposite pattern in anxious MDD. As predicted, symptom remission was associated with increases in self-esteem and this correlated with the frequency with which participants employed the psychological strategies in daily life.

**Conclusions:**

These findings suggest that self-blame-rebalance neurofeedback may be superior over a solely psychological intervention in non-anxious MDD, although further confirmatory studies are needed. Simple self-guided strategies tackling self-blame were beneficial, but need to be compared against treatment-as-usual in further trials. https://doi.org/10.1186/ISRCTN10526888

## Introduction

Functional MRI neurofeedback provides individuals with information about the neural activity that is outside of their awareness and thus overcomes one limitation of cognitive therapy, which mainly relies on conscious self-reflection (Beck, Rush, Shaw, & Emery, 1979). Here, we report the first clinical trial of a neurofeedback intervention to tackle self-blaming biases in symptomatic major depressive disorder (MDD). Recent findings confirm blame attributional models of MDD (Abramson, Seligman, & Teasdale, 1978), highlighting the importance of overgeneralised self-blame and the resulting feelings of worthlessness, overgeneralised guilt and self-disgust for MDD (Green, Moll, Deakin, Hulleman, & Zahn, 2013; Zahn et al., 2015a, 2015b). Self-criticism is also a central target of cognitive behavioural therapy (Beck et al., 1979). So far, functional magnetic resonance imaging (fMRI) neurofeedback interventions for symptomatic MDD have been designed, however, on the basis of a model that proposes an overall increase in negative and reduction in positive emotions (Watson, Clark, & Carey, 1988) rather than self-blame-selective increases in negative emotions.

The neural signature of self-blaming biases in MDD has been elucidated in a series of studies. Using fMRI, abnormal functional connectivity between the right superior anterior temporal lobe (rSATL) and the anterior subgenual cingulate cortex was associated with overgeneralised self-blaming emotions in remitted MDD cross-sectionally (Green, Lambon Ralph, Moll, Deakin, & Zahn, 2012). Increased self-blame-selective functional connectivity in remitted MDD between the rSATL and posterior subgenual cortex (SC, BA25) predicted risk of future major depressive episodes (MDEs) over the subsequent year (Lythe et al., 2015). We provided the technical proof-of-concept that changes in self-blame-selective rSATL-subgenual cingulate cortex connectivity can be detected and fed back to healthy control (HC) participants during real-time fMRI (Sato et al., 2013). Further, a recent double-blind, randomised controlled clinical trial (RCT) demonstrated that people with remitted MDD can use fMRI neurofeedback to successfully rebalance self-blame-related rSATL-subgenual cingulate cortex patterns in a single training session, thereby increasing their self-esteem (Zahn et al., 2019). This work builds on extensive evidence for the pathophysiological importance of SC networks in MDD (Drevets & Savitz, 2008; Dunlop et al., 2017; Price & Drevets, 2010; Ressler & Mayberg, 2007; Siegle, Carter, & Thase, 2006).

Only a few studies to date have investigated fMRI neurofeedback in current MDD (Hamilton et al., 2016; Young et al., 2017a, 2018a, 2018b; Yuan et al., 2014; Zotev et al., 2016; Zotev, Phillips, Yuan, Misaki, & Bodurka, 2014), and even fewer studies have investigated clinical outcomes (Linden et al., 2012; Mehler et al., 2018; Young et al., 2014, 2017b). Linden et al.'s pioneering non-randomised study (*n* = 16) applied fMRI neurofeedback to increase activation in brain areas identified using a functional localiser for positive emotions (Linden et al., 2012), whereas another non-randomised study used neurofeedback training (*n* = 21) to enhance amygdala response during the recall of positive autobiographical memories (Young et al., 2014). Both studies were promising and led to two more recent RCTs comparing active *v.* control fMRI neurofeedback, with one trial (*n* = 36) showing the superiority of the active intervention (Young et al., 2017a) and the other trial showing equivalent benefits for both trial arms (*n* = 32; (Mehler et al., 2018).

The current NeuroMooD RCT examined the clinical potential and feasibility of a novel self-guided psychological intervention which incorporated cognitive strategies to tackle self-blame with and without additional fMRI neurofeedback in insufficiently recovered patients with MDD. The following hypotheses were investigated:

Hypothesis 1 (pre-registered main hypothesis): Patients undergoing neurofeedback training will show reduced depressive symptoms, decreased self-blame and increased self-worth when compared with the psychological intervention group.

Hypothesis 2 (specific secondary): Patients undergoing neurofeedback training will show decreased self-blame-selective hyper-connectivity between the rSATL and the posterior SC post-treatment compared to pre-treatment (one of our pre-registered secondary outcome measures).

Hypothesis 3 (specific secondary): Decreased self-blame-selective hyper-connectivity between the rSATL and the posterior SC region will be associated with a reduction in depressive symptoms in MDD.

## Method

This ISRCTN pre-registered (#10526888) RCT was approved by the Health Research Authority & NRES Committee London – Camberwell St Giles (REC reference: 15/LO/0577) and all trial participants gave written informed consent. Researchers involved in the conduction of this clinical trial affirm that study procedures complied with the ethical principles, standards and national and institutional guidelines for clinical trials and research involving human subjects and with the Helsinki Declaration of 1975, as revised in 2008. Fully anonymised data will be made available via King's College London's data repository. Reporting conforms with the CRED-nf guidelines ((Ros et al., 2020), see online Supplementary Materials).

### Trial design

After a modification to our initial protocol (see online Supplementary Methods), a single-blind, RCT design was used, and after a baseline assessment (visit 1) participants were randomised to two parallel treatment groups, each comprising three intervention visits (visits 2, 3 & 4). Clinical outcomes were assessed after treatment completion (visit 5). Regardless of the intervention group, treatment sessions were scheduled 7–13 days apart.

### Randomisation method

Our Clinical Trials Unit randomised participants using an automated computerised system and a stratified block design with randomly varying block sizes, deploying two stratification factors: gender (female/male) and categorised baseline scores of the Beck Depression Inventory-II (BDI-II; (Beck, Steer, & Brown, 1996), see online Supplementary Methods).

### Recruitment and reimbursement of participants

We recruited participants from September 2016 to December 2017, advertising primarily online, as well as via recruitment circulars, and presenting to self-help groups at scheduled member meetings. Participants received compensation (online Supplemental Methods).

### Inclusion and Exclusion criteria

We included right-handed participants (to ensure homogenous responses to the right hemispheric treatment target), ⩾18 years old, proficient in English, with recurrent MDD according to the Diagnostic and Statistical Manual of Mental Disorders-5 (APA, 2013), who in their current episode of ⩽12 months had insufficiently responded to ⩾1 pharmacological/psychotherapeutic treatment or were not amenable to standard treatments. They had to report significantly impairing or bothering symptoms [Psychiatric Status Rating Scale ⩾3, (Keller et al., 1987)] over the past 2 weeks and no change in antidepressant medications and symptoms in the 6 weeks preceding visit 1, as well as agreeing to keep medications unchanged throughout the trial. People currently undergoing psychotherapy were excluded. Main exclusion criteria consisted of MRI contraindications, a history of schizophreniform or hypomanic symptoms or other relevant psychiatric or medical co-morbidity (see online Supplementary Methods).

### Eligibility assessment

Following the clinical evaluation (*n* = 71), 43 participants were randomised into the study, of which *n* = 35 participants completed the trial (see Consort Flowchart in online Supplementary Fig. S3, clinical assessment methods and experimental tasks are described in online Supplementary Methods).

### Intervention procedures

Both intervention arms entailed the same self-guided psychological intervention further described below during sessions and whenever self-blaming thoughts arose in participants' daily life. Before the first intervention session (see online Supplementary Table S1, online Supplementary Methods), participants were asked to provide two cue words, prompting them to remember two autobiographical events associated with strong feelings of self-blame/guilt and two cue words linked to two events associated with blame or indignation/anger towards others whilst feeling low levels of self-blame.

### Psychological intervention

Participants were instructed to select the most suitable out of the following strategies to help them manage their feelings: ‘Think about’: (1) ‘why you might not have been in control over the outcome of the event’, (2) ‘why you might not be responsible for the outcome of the event’, (3) ‘why the consequences for others might not be so bad’, (4) ‘making up for things or apologising’, (5) ‘the other person forgiving you’, (6) ‘forgiving yourself’. If preferred, they could also develop their own strategies.

These strategies were based on (1) attribution theory which highlights the importance of locus of control for self-blame (Abramson et al., 1978), on (2) omnipotent responsibility associated with depressogenic forms of guilt (O'Connor, Berry, Weiss, & Gilbert, 2002), on (3) neurocognitive models of self-blame, implicating representations of future consequences as important to guilt-proneness (Zahn, de Oliveira-Souza, & Moll, 2020), on (4) the associations of reparative action tendencies with adaptive forms of guilt (Tangney, Stuewig, & Mashek, 2007), as well as on the focus on (5) forgiveness and (6) self-kindness as thematised in compassion-focused therapy (Gilbert, 2010). Further details about the timing of the intervention which was matched to the neurofeedback procedure are described in online Supplementary Methods.

### fMRI neurofeedback intervention

Each session comprised four runs. The first and fourth runs (204 volumes each; 408 s duration) were identical and served to determine pre- and post-neurofeedback effects (online Supplementary Fig. S1). They consisted of four self-blame blocks (15 volumes each) and four other-blame blocks (15 volumes each), interspersed with eight mental subtraction condition blocks (10 volumes each). During the subtraction blocks, participants were asked to mentally subtract seven from a 3-digit number (e.g. 101, 102).

The neurofeedback training runs (runs 2&3: 212 volumes each; 424 s duration) were identical and consisted of four self-blame blocks (42 volumes per block), interspersed with four mental subtraction condition blocks (10 volumes each). An upward and downward moving thermometer scale (online Supplementary Fig. S2) was displayed to provide visual feedback on how successful participants were in modifying their brain correlation patterns (see below). The thermometer scale appeared in the form of a colour bar that could reach different levels. Participants were instructed to think about the particular autobiographical scenario triggered by the display of the previously agreed cue word and to try and bring up the level to the top of the thermometer scale by choosing a psychological strategy from the list they had been provided with before the scanning session. Mental subtraction blocks were used to distract participants from the emotionally charged autobiographical memories, in addition to minimising resting-state activity in the posterior SC region (Moll et al., 2014).

### fMRI neurofeedback method

Image acquisition details are described in the online Supplementary Methods. The FRIEND fMRI neurofeedback software (Basilio et al., 2015; Sato et al., 2013) was used (file version 1.0.0.257, online Supplementary Figs S1 and S2). FRIEND has previously been validated for correlation feedback in patients with MDD (Zahn et al., 2019).

Methodological specifications and validation of FRIEND (https://www.nitrc.org/projects/friend) which is a freely available toolbox for Oxford University's FSL package (https://fsl.fmrib.ox.ac.uk/fsl/) have been described elsewhere (Basilio et al., 2015; Moll et al., 2014; Sato et al., 2013; Zahn et al., 2019). In the NeuroMooD trial, FRIEND provided ROI-based fMRI neurofeedback alongside executing fundamental pre-processing of the fMRI data in real-time. Facilitated by native FSL code, FRIEND performed motion correction using MCFLIRT, spatial smoothing with a Gaussian Kernel (FWHM = 6 mm) (Zahn et al., 2019). Signal-level normalisation was performed by subtracting the mean value of the voxel signals within the ROI over the entire preceding subtraction condition block from the current echo-planar images belonging to the self-blame or other-blame condition block, which minimises local signal trends (Zahn et al., 2019).

The rSATL ROI (consisting of the same region used as a seed region in our previous studies (Green et al., 2012)) and posterior SC ROI [consisting of the BA 25 cluster whose self-blame-selective hyper-connectivity was associated with recurrence risk (Lythe et al., 2015)] were pre-defined, warped from MNI space into subject structural space and ultimately back-transformed into native space, using the inverse of the transformation algorithm of FSL FLIRT (affine, 12 parameters). During run 1, a general linear model was used to generate a t-map to derive the most activated 50% of voxels, which were selected in the native space ROI, as determined on contrast images for self-blame *v.* subtraction in the rSATL ROI, and self-blame *v.* other-blame in the posterior SC ROI. These voxels were used to extract the average signal for the subsequent fMRI neurofeedback training. The first five volumes of each emotional block were discarded (Zahn et al., 2019). A moving target correlation algorithm was employed by using a sliding time window based on the last 10 volumes, updated every two seconds (i.e. for each volume). The level of the colour bar of the visual feedback signal was determined on the basis of the size of the Pearson correlation coefficient measured over the last 10 volumes [weighted by a sigmoid function,(Zahn et al., 2019)] in relation to the minimum and maximum.

### Statistical power and offline analyses

Statistical power was calculated using G*POWER (Faul, Erdfelder, Buchner, & Lang, 2009) and required a sample size of *n* = 34 participants to achieve 85% power at *p* = 0.05, 2-sided (*t* test). This calculation was based on a more conservatively estimated effect size (*d* = 1.06) than the effect size (*d* = 1.5 for the reduction in the 17-item Hamilton Depression Rating Scale score in the neurofeedback group) reported in a previous fMRI neurofeedback study in MDD (Linden et al., 2012), but may not have been conservative enough given lower effect sizes reported in other neurofeedback studies (Zaehringer et al., 2019). The enrolment target was *n* = 45 overall and *n* = 36 completers, very slightly above the *n* = 35 completers we achieved. To determine precise effect sizes, a larger study is needed which should include at least 70 participants in order to estimate the pooled standard deviation for continuous outcomes in RCTs (Teare et al., 2014). 22 participants were randomised to the fMRI neurofeedback group and 21 to the psychological intervention group (see online Supplementary Table S2 for clinical, and online Supplementary Table S3 for demographic characteristics). 8 participants withdrew or were excluded during the duration of the trial, leading to a final of *n* = 35 for the primary analysis (see online Supplementary Fig. S3 for the Consort Flow Diagram).

After seeking statistical advice (K.G., E.C.), group-level analyses of primary and secondary outcomes using SPSS24 (https://www.ibm.com/analytics/spss-statistics-software), comparing pre- and post-treatment effects (visit 1 *v.* visit 5), were obtained using the constrained longitudinal analysis model (cLDA), which is identical to an analysis of variance on final outcomes whilst covarying baseline measures for complete data, but is preferable when there are cases lost to follow-up (Coffman, Edelman, & Woolson, 2016)). The alpha level was set to *p* = 0.05, two-tailed. As in our previous paper (Zahn et al., 2019) at the individual subject level, linear regression coefficients for the slope of z-transformed rSATL signal time-course as the predictor of z-transformed SC signal time-course in each condition (self-blame, other-blame) in the pre- and post-training acquisition as the outcome variables were derived from a general linear model for each subject by modelling the interaction of z-transformed rSATL signal time-course with two factors: condition (self-blame, other-blame) and time (pre-, post-training). The z-transformation was undertaken to obtain standardised regression coefficients. Cohen's d effect sizes were computed for each regression coefficient using the formula: 2 × t-value/square root (df) (Rosenthal & Rosnow, 1991).

Where cLDA was not applicable, intervention group comparisons were performed using non-parametric tests. A repeated-measures ANOVA was chosen in the analysis of regression coefficients for z-transformed rSATL and posterior SC signals in the self-blame and other-blame conditions. Secondary data analyses of the anxious distress subtype of MDD were conducted using univariate GLM analysis. As analyses were either hypothesis-driven or exploratory (secondary outcome measures in the feasibility trial), *p* value adjustments to correct for multiple comparisons were not carried out (Feise, 2002).

## Results

### Pre-registered primary outcome measure

A significant improvement, irrespective of the treatment group, was found on the pre-registered primary outcome measure, the BDI-II (Beck et al., 1996). BDI-II scores showed an overall reduction of 46.1% after treatment, which corresponds to a baseline mean = 29.1 points [standard deviation (s.d.) = 8.66, *n* = 43] and a post-intervention mean of 15.7, (s.d. = 9.75, *n* = 35). The cLDA analysis demonstrated a strong main effect of treatment irrespective of the intervention group [post-treatment BDI-II mean of 13.39, standard error [s.e.] = 2.74, degrees of freedom (df) = 75, *t* = 4.89, *p* < 0.001, 95% confidence interval (CI) 7.93–18.85, Cohen's *d* = 1.13). Contrary to our hypothesis, however, the analysis showed no effect of intervention group on the primary outcome measure (BDI-II mean difference = 0.07, s.e. = 3.17, df = 75, *t* = 0.02, *p* = 0.984, 95% CI −6.26 to 6.3, Cohen's *d* = 0.00) with identical post-treatment BDI-II scores in both groups (psychological intervention: *n* = 16; *M* = 15.75, s.d. = 9.75; fMRI neurofeedback: *n* = 19; *M* = 15.68, s.d. = 10.02). Thus, both interventions were shown to be equally beneficial with 56% treatment responders in the psychological intervention group and 58% treatment responders in the fMRI neurofeedback group (Numbers Needed to Treat for response to neurofeedback *v.* psychological intervention was 50). Treatment response was defined as an improvement of ⩾50% on the defined outcome measure.

## Pre-registered secondary outcome measures

### Measures of symptoms, self-esteem and self-blame

Intervention group comparisons on the pre-registered secondary outcome measures are presented in Tables 1–3. In both intervention groups, there were significant improvements on measures of depressive symptoms, including the Montgomery-Åsberg Depression Rating Scale [MADRS, (Montgomery & Åsberg, 1979)], Quick Inventory of Depressive Symptomatology [QUIDS-SR16, (Rush et al., 2003)] and the depression-dejection subscale of the Profile of Mood States (POMS) Scale (McNair, Lorr, & Doppleman, 1971). Clinical global impression scales indicated a median of 2 in both groups when judged by the blinded observer (i.e. much improved). Moreover, MDD patients' self-esteem increased significantly post- *v.* pre-treatment, regardless of the intervention group. There was a clear reduction in self-blaming emotions in both groups based on the autobiographical memory ratings and when assessed by the blinded rater using a semi-structured interview designed to assess these emotions [Table 5, (Zahn et al., 2015b)]. Nevertheless, and against our hypothesis, the neurofeedback intervention was not superior over the solely psychological intervention on any of the pre-registered secondary outcome measures.

**Table 1.** Intervention group comparisons on pre-registered continuous secondary outcome measures

	PRE-INTERVENTION		POST-INTERVENTION				cLDA EFFECT OF TIME						cLDA EFFECT OF GROUP					
	PSYCH&NFB		PSYCH		NFB		BASELINE v. FINAL						PSYCH v. NFB					
MEASURE [sample size]	*M*	s.d.	*M*	s.d.	*M*	s.d.	diff [df]	s.e.	*p*	95% CI	*t*	*d*	diff [df]	s.e.	*p*	95% CI	*t*	*d*
MADRS [PRE: 43; POST: 35]	22.84	6.97	15.56	6.38	14.37	6.55	7.27 [75]	1.98	0.00*	3.33 to 11.22	3.68	0.85	1.19 [75]	2.29	0.60	−3.37 to 5.76	0.52	0.12
QUIDS-SR16 [PRE: 43; POST: 35]	16.79	6.53	10.88	6.53	10.16	4.41	5.92 [75]	1.67	0.00*	2.58 to 9.25	3.53	0.82	0.72 [75]	1.94	0.71	−3.15 to 4.58	0.37	0.09
POMS 2 Depression-Dejection Scale [PRE: 43; POST: 35]	10.91	4.47	7.81	4.59	7.21	5.67	3.09 [75]	1.41	0.03*	0.29 to 5.90	2.20	0.51	0.60 [75]	1.63	0.71	−2.65 to 3.85	0.37	0.09
Rosenberg Self-Esteem Scale [PRE: 43; POST: 35]	20.60	3.49	24.19	4.15	24.58	5.26	−3.59 [75]	1.20	0.00*	−5.98 to −1.18	−2.97	−0.69	−0.39 [75]	1.40	0.78	−3.17 to 2.39	−0.28	−0.06
Implicit Self-Blame Contempt-Anger [PRE: 41; POST: 35]	0.00	39.00	−0.28	0.36	−0.05	0.39	0.29 [73]	0.11	0.01*	0.06 to 0.51	2.53	0.59	−0.23 [73]	0.13	0.08	−0.49 to 0.03	−1.8	−0.42
Implicit Self-Blame Contempt-Anxiety [PRE: 38; POST: 35]	0.32	38.00	0.24	0.42	0.24	0.47	0.08 [70]	0.12	0.50	−0.16 to 0.32	0.68	0.16	0.00 [70]	0.13	0.98	−0.27 to 0.26	−0.03	−0.01
Agency-Incongruent Self-Blame [PRE: 43; POST: 35]	15.25	16.85	10.76	14.97	10.23	17.59	4.48 [75]	4.88	0.36	−5.25 to 14.21	0.92	0.21	0.53 [75]	5.66	0.93	−10.7 to 11.80	0.09	0.02

* = significant at *p* = 0.05, 2-sided. Between-group Cohen's d scores were computed from t-values and degrees of freedom (Rosenthal & Rosnow, 1991) of the differences between groups. Mean differences and 95% CIs were taken from cLDA models for differences between post- v. pre-training. Implicit self-blame was measured using the Brief Implicit Association Test. Agency-incongruent self-blame was measured using the modified version of the Value-related Moral Sentiment Task. PSYCH = psychological intervention group, NFB = fMRI neurofeedback group. CI, confidence interval; *M*, mean; s.d., standard deviation; s.e., standard error; diff, difference of means; df = degrees of freedom; *d* = Cohen's d.

**Table 2.** Intervention group comparisons on pre-registered non-continuous secondary outcome measures assessed post-intervention only

MEASURE [sample size]	MANN-WHITNEY U	ASYMP. SIG.	EXACT SIG.	MEDIAN		RANGE		MINIMUM		MAXIMUM	
				PSYCH	NFB	PSYCH	NFB	PSYCH	NFB	PSYCH	NFB
Observer-Rated CGI Post-Intervention [35; NFB: 19; PSYCH: 16]	129.00	0.376	0.461	2.00	2.00	2.00	3.00	2.00	2.00	4.00	5.00
Participant-Rated CGI Post-Intervention [35; NFB: 19; PSYCH: 16]	139.50	0.658	0.683	2.50	2.00	4.00	4.00	1.00	1.00	5.00	5.00
Withdrawal Rates throughout trial [43; NFB: 22; PSYCH: 21]	218.50	0.635		0.00	0.00	1.00	1.00	0.00	0.00	1.00	1.00
Withdrawal Rates after first treatment session [43; NFB: 22; PSYCH: 21]	221.00	0.582		0.00	0.00	1.00	1.00	0.00	0.00	1.00	1.00
Adverse Events throughout trial [43; NFB: 22; PSYCH: 21]	208.00	0.352		0.00	0.00	1.00	1.00	0.00	0.00	1.00	1.00
Adverse Events after first treatment session [43; NFB: 22; PSYCH: 21]	219.50	0.527		0.00	0.00	1.00	1.00	0.00	0.00	1.00	1.00

Asymp. Sig., 2-tailed; Exact Sig., 2*(1-tailed Sig.). PSYCH, psychological intervention group; NFB, fMRI neurofeedback training group.

**Table 3.** Intervention group comparisons on pre-registered non-continuous secondary outcome measures collected pre- and post-intervention

	PRE-INTERVENTION						POST-INTERVENTION						NON-PARAMETRIC COMPARISONS					
	PSYCH			NFB			PSYCH			NFB			BETWEEN-GROUP FOR POST-PRE			POST v. PRE		
MEASURE [sample size]	Md	Min	Max	Md	Min	Max	Md	Min	Max	Md	Min	Max	χ^2^ Median Test	*n*	*p*	Wilcoxon-Standardized	*n*	*p*
Participant-Rated Self-Blame [PRE:39; POST:35]	8.5	7.0	10.0	8.5	4.5	10.0	5.0	2.0	9.0	4.0	1.0	9.0	0.12	34	0.73	−4.84 (reduced in 32/35)	35	<0.0001
Observer-Rated Self-Blame [PRE:43; POST:35]	2.0	0.0	7.0	2.0	0.0	7.0	1.0	0.0	4.0	1.0	0.0	5.0	0.02	35	0.90	−3.97 (reduced in 27/35)	35	<0.0001

Participant-rated self-blame scores are based on the mean of two autobiographical events per subject; Observer-rated self-blame was defined as per trial register as the sum of all self-blaming emotion scores (guilt/shame, self-directed anger, and self-disgust/contempt/hate/loathing, each of these 3 emotion scores is scored on a scale from 0 = absent, 1 = mild/minimal, 2 = moderate, 3 = overgeneralised and severe, resulting in a minimum sum score of 0 and maximum sum score of 9) are based on the moral emotion addendum of the AMDP Psychopathology Interview questions on depression(Faehndrich & Stieglitz, 2007; Zahn et al., 2015)). PSYCH = psychological intervention group, NFB = fMRI neurofeedback group. Mdn = Median, Min = Minimum, Max = Maximum, df = degrees of freedom. For means and standard deviations for the self-blame measures, please see online Supplementary Table S6.

### Adverse events and withdrawal rates

Notably, fMRI neurofeedback training as well as the solely psychological intervention were found to be safe and showed a high retention rate of participants. No significant differences between interventions emerged regarding adverse events or withdrawal rates throughout the trial following randomisation or specifically, between the first treatment session and trial completion (Table 4). There were no serious or medically important adverse events (see online Supplementary Results). Throughout the trial, seven participants withdrew their consent and ended their participation in the study, four prior to the first day of the intervention and three at different time points following their first treatment session (see online Supplementary Results).

**Table 4.** Post-training v. pre-training comparison of pre-registered rSATL – posterior SC connectivity on fMRI in neurofeedback group only

				PRE-TRAINING		POST-TRAINING		REPEATED MEASURES ANOVA					
				NFB		NFB		TIME		CONDITION		TIMExCONDITION	
MEASURE [sample size]				*M*	s.d.	*M*	s.d.	*F*	*p*	*F*	*p*	*F*	*p*
Guilt: rSATL-SC regression coefficient: Cohen's d				0.29	0.54	0.16	0.37	0.29	0.87	0.43	0.52	6.40	0.02*
Other-blame: rSATL-SC regression coefficient: Cohen's d				0.15	0.46	0.24	0.25						

* = significant at *p* = 0.05, 2-sided. NFB, fMRI neurofeedback group; rSATL-SC, right superior anterior temporal lobe – posterior subgenual cortex. *n* = 18 with complete data.

### fMRI neurofeedback group connectivity changes

**Fig. 1.**
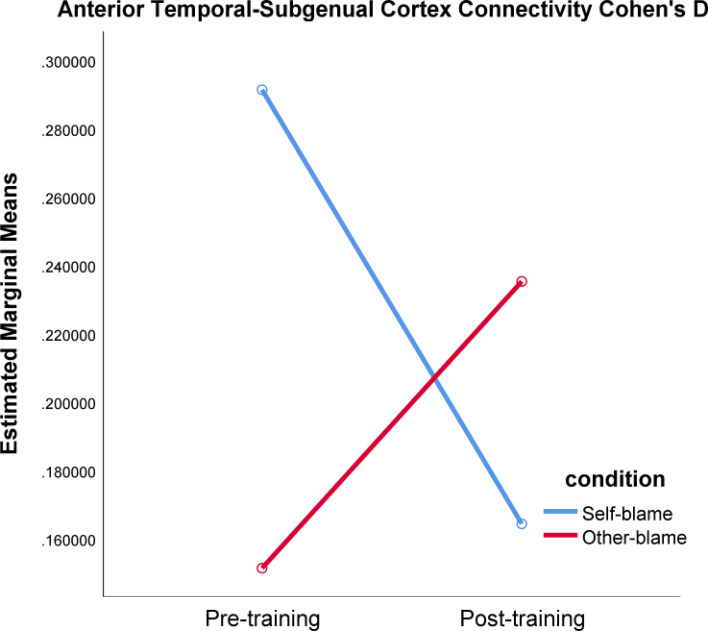
Relative change in functional connectivity between rSATL and posterior SC in the self-blame and other-blame condition, measured as Cohen's D for regression coefficient means for time series pre- and post-fMRI neurofeedback training, comparing the first and final treatment session. See Table 4 for statistics.

As predicted, a significant training-induced reduction in connectivity between the rSATL and posterior SC was detected in the self-blame condition relative to other-blame, as reflected in a significant time by condition interaction (Fig. 1, Table 4). Inconsistent with our prediction, this decrease was not found to be significant for the self-blame condition itself (*t* = −0.89, df = 17, *p* = 0.387; *n* = 18, mean difference between conditions = −1.27, 95% CI −0.43 to 0.18). Interestingly, as self-blame-related connectivity successfully reduced relative to other-blame post-treatment, other-blame-related connectivity between the rSATL and posterior SC was observed to increase (mean difference = 0.09 post- *v.* pre-fMRI neurofeedback training; Table 4). This finding, however, was not significant itself (*t* = 0.68, df = 17, *p* = 0.504, 95% CI −1.76 to 3.43).

In addition to applying the cLDA model, the intention-to-treat (ITT) approach was chosen and compared with the per-protocol analyses, using the χ^2^ test to analyse the association between the intervention group and treatment response on the primary outcome measure (BDI-II). The ITT analysis included data of all randomised participants regardless of their adherence or withdrawal subsequent to randomisation (Fisher, 1990). Here, participants who withdrew from the study or did not complete the trial were treated as non-responders. No relationship was found between the intervention group and treatment response χ^2^(1, *N* = 43) = 0.029, *p* = 0.864. Similarly, when only including completers, no association between the intervention group and treatment response was found χ^2^(1, *N* = 35) = 0.046, *p* = 0.830. The results of these additional analyses confirmed the per-protocol analyses using cLDA.

Throughout all treatment sessions and active neurofeedback runs, participants were able to successfully bring down the level of self-blame-associated correlations as reflected in an average neurofeedback thermometer level of around 50% (online Supplementary Fig. S3). This is remarkable considering that FRIEND implements a moving target algorithm potentially making it more difficult to control the neurofeedback thermometer as connectivity between the rSATL-posterior SC successively reduces with training (for further analyses of how connectivity changed over the 3 treatment sessions, see online Supplementary Fig. S5).

## Exploratory secondary data analysis

### Major depressive disorder with and without anxious distress

**Fig. 2.**
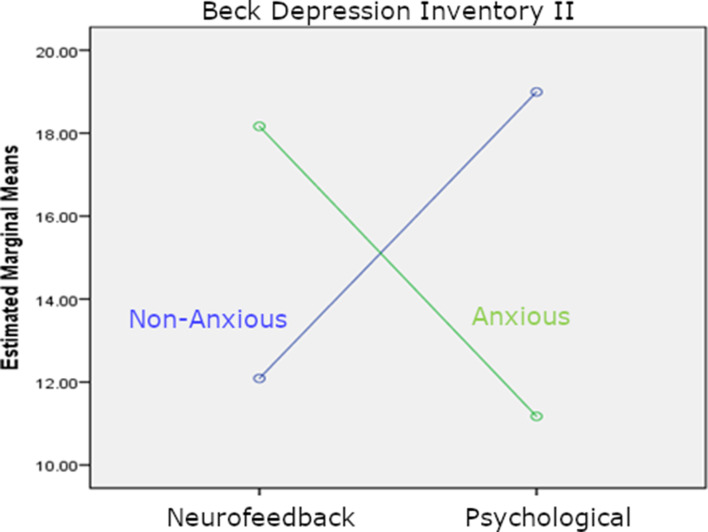
The results of a secondary analysis are displayed which stratified our primary outcome by anxious distress features, the most frequent major depressive disorder (MDD) subtype in our trial (*n* = 21 out of *n* = 35, Structured Clinical Interview for DSM5). Plotted are post-treatment BDI-II estimated marginal means of MDD patients with and without anxious distress in both treatment groups (fMRI neurofeedback group: *n* = 19; psychological intervention group: *n* = 16). Covariates appearing in the model are evaluated at the estimated baseline BDI-II value of 28.6 points.

A novel DSM-5-specifier for MDD with anxious distress was found to be most common in our sample (online Supplementary Table S2). A univariate GLM showed a significant interaction between the treatment group and anxious distress (*F*_(1,30)_ = 4.98, *p* = 0.033, *η*p^2^ = 0.14). This interaction was due to a better response to the neurofeedback-enhanced intervention in non-anxious (83% of patients halving their BDI-II scores) *v.* anxious patients (46%) and a better response to the solely psychological intervention in anxious (75%) *v.* non-anxious (38%) patients. There was no significant main effect of the MDD anxious/non-anxious subtype (*F*_(1,30)_ = 0.78, *p* = 0.782, *η*p^2^ = 0.003), nor the main effect of treatment group (*F*_(1,30)_ = 0, *p* = 0.989, *η*p^2^ = 0; Fig. 2).

In online Supplementary Table S4, we showed that anxious and non-anxious MDD patients were well matched on general demographic and clinical variables. As one would predict from the association of anxiety and irritability/anger in DSM-5 diagnostic criteria for some anxiety disorders, we found higher levels of current anger towards others in the anxious compared with the non-anxious MDD group as measured on our psychopathological interview. Interestingly, there was no subtype difference in the sum of self-blaming emotions score computed as a secondary outcome measure using this interview.

### Change in connectivity on fMRI, self-esteem and engagement in treatment

Based on previous findings, where measures of self-esteem correlated with changes in functional connectivity between the rSATL and the anterior subgenual cingulate after fMRI neurofeedback training (Zahn et al., 2019), non-parametric correlation analyses were conducted to explore this pattern in the fMRI neurofeedback group (online Supplementary Table S5). Notably, no such correlation was found; a change in connectivity between the rSATL and the posterior SC for self-blame relative to other-blame was not associated with an increase in self-esteem in the patient group. Interestingly, however, improvement in depression scores correlated with an increase in self-esteem. Similarly, a positive correlation was found between increased self-esteem and engagement in treatment as assessed by the summed frequency of use of treatment-specific psychological strategies throughout the study in both intervention groups.

## Discussion

This RCT in MDD investigated the clinical potential of a self-guided psychological intervention to rebalance self-blame with and without being guided by fMRI neurofeedback. We hypothesised that patients randomised to fMRI neurofeedback would show a reduction in depressive symptoms and self-blame while exhibiting an increase in self-worth compared to the solely psychological intervention. Furthermore, we predicted that patients undergoing fMRI neurofeedback training would show a decreased functional connectivity between the rSATL and the posterior SC post-treatment compared with pre-treatment. Decreased functional connectivity between the rSATL and posterior SC region was further predicted to be associated with a reduction in depressive symptoms in the fMRI neurofeedback group.

The results demonstrated that both interventions were safe, with no medically important or serious adverse events occurring in either group. There was a strong effect size for patients' improvement on self-rated and observer-rated depression measures, with response rates above 55% in both intervention groups. The safety and overall clinical benefits of our fMRI neurofeedback intervention are in keeping with previous studies (Linden et al., 2012; Young et al., 2014, 2017a; Mehler et al., 2018; Young et al., 2017a). This is particularly remarkable as NeuroMooD asked participants to engage with negative rather than positive emotions as in previous studies (Young et al., 2014, 2017a, 2018a, 2018b). Contrary to our first hypothesis, no difference was found between the fMRI neurofeedback and the psychological intervention group on the primary outcome measure (BDI-II). Our second prediction was confirmed as the fMRI neurofeedback training resulted in a decrease in functional connectivity between the rSATL and the posterior SC for self-blame relative to other-blame. Contrary to our third hypothesis, no relationship was found between connectivity changes and the changes in depressive symptoms after three sessions of fMRI neurofeedback training.

Various considerations need to be taken into account why no intervention group differences were found. One possibility is that the improvements observed in both intervention groups were due to spontaneous remission or placebo-like effects instead of being the result of treatment. This is possible, yet unlikely to be the only explanation as the placebo response rate in MDD is generally found to be lower, usually, around 30% (Walsh, Seidman, Sysko, & Gould, 2002), which is well below the >55% response rate demonstrated in both treatment groups in our trial. Furthermore, NeuroMooD reduced the risk of spontaneous remission by including MDD patients with early treatment resistance. Lastly, the frequency of how often participants used the psychological strategies between treatment visits was found to positively correlate with an increase in self-esteem in both intervention groups, which further argues against spontaneous remission as the sole explanation for the observed findings. Apart from a lack of power, another possible explanation why fMRI neurofeedback did not show superiority over the psychological intervention is that neurofeedback provided no added therapeutic value compared to the already strong effects of the psychological aspects of the intervention. While this cannot be ruled out, the secondary analysis suggests that the neurofeedback training was superior to the solely psychological intervention in non-anxious MDD patients.

The observed neurofeedback training-induced reduction in rSATL-SC connectivity for self-blame relative to other-blame demonstrates that patients were able to successfully modulate their brain connectivity as guided by the feedback. The lack of association between connectivity changes and improvement in depressive symptoms is in keeping with the limited added benefit of fMRI neurofeedback overall as the majority of patients were of the anxious distress subtype. One reasonable explanation for this finding is that the fMRI target may be irrelevant for most anxious MDD patients. This would be in keeping with our finding of anger towards others being a distinctive feature of the anxious distress group which showed highly consistent self-blaming emotions at the same time. Future studies in larger samples are needed to investigate whether this co-existence of self- and other-blaming emotions in anxious MDD is also reflected in a lack of self-blame-selective neural changes. Another implication is that previous neurofeedback approaches which were developed for enhancing positive emotions overall (Mehler et al., 2018; Young et al., 2017b) may be more suitable for anxious MDD, which calls for stratified allocation to different neurofeedback targets in future trials.

The observed fMRI neurofeedback training-induced reduction in functional connectivity between the rSATL and the posterior SC for self-blame relative to other-blame demonstrates that MDD patients were able to successfully modulate their brain connectivity as guided by the fMRI neurofeedback signal. The lack of association between functional connectivity changes and improvement in the severity of depressive symptoms is in keeping with the limited added benefit of fMRI neurofeedback overall as the majority of patients were of the anxious distress subtype. This suggests that the neural fMRI target may be irrelevant for the anxious distress subtype of MDD; a hypothesis that needs to be examined in future larger studies.

### Limitations

On a more cautionary note, the study might have been underpowered and, therefore, unable to detect a clinically meaningful difference between the two intervention groups. The effect sizes for non-superiority of the fMRI neurofeedback group, however, were so small that even a large sample would have been unable to find differences between groups. Furthermore, the trial's sample size was comparable to other RCTs investigating fMRI neurofeedback in MDD (Mehler et al., 2018; Young et al., 2017a). Another limitation was that we lacked longer-term follow-up outcome data which may have proven neurofeedback to be superior as suggested by some studies(Rance et al., 2018). Our secondary analyses stratifying for subtype were limited by not being pre-registered and by the relative scarcity of non-anxious MDD patients and thus need reproducing in a larger sample. Finally, the NeuroMooD study lacked a neurofeedback control arm; this was deliberate in order to probe the clinical usefulness of neurofeedback relative to a cheaper intervention. Had we found fMRI superiority over the control intervention, this would have led to some difficulties in ruling out non-specific placebo-like effects of neurofeedback. The fact that there was no superiority of neurofeedback overall in our study, however, suggests that being in a scanner environment did not itself have strong placebo-like effects. Furthermore, control neurofeedback interventions are difficult to design and interpret. Young et al. used the left intraparietal sulcus signal in the control neurofeedback condition which is not relevant for recalling positive emotions, the task given to participants (Young et al., 2017a). This mismatch between neurofeedback signal and psychological instructions could have contributed to the inferiority of the control intervention by distracting participants from the psychological task they were given.

## Conclusion

Both trial interventions resulted in a 46% reduction in symptoms in MDD patients who had insufficiently responded to standard treatment. Although a contribution of non-specific effects cannot be ruled out, it is likely that the psychological intervention had specific therapeutic effects. Our secondary analysis suggests that self-blame-selective fMRI neurofeedback training is of superior benefit in non-anxious MDD patients compared with the solely psychological intervention, although this needs further confirmation in a larger sample.

## Supplementary material

For supplementary material accompanying this paper visit https://doi.org/10.1017/S0033291721004797. click here to view supplementary material
