# Supplementary material for: Self-blame in major depression: a randomised pilot trial comparing fMRI neurofeedback with self-guided psychological strategies
Source: Psychol Med. 2021 Dec 2;53(7):2831–41. doi: 10.1017/S0033291721004797 (PMC10235657; doi:10.1017/S0033291721004797)
Supplement: Supplementary file 1 [file S0033291721004797sup001.docx]

**SUPPLEMENTARY MATERIAL**

**Self-blame in major depression: a randomised pilot trial comparing fMRI neurofeedback with self-guided psychological strategies**

Tanja Jaeckle^1^, Steven C.R. Williams^2^, Gareth J. Barker^2^, Rodrigo Basilio^3^, Ewan Carr^4^, Kimberley Goldsmith^4^, Alessandro Colasanti^1^, Vincent Giampietro^2^ _,_ Anthony Cleare^1^, Allan H. Young^1,5^, Jorge Moll^3^ and Roland Zahn^1,3,5*^

*^1^ Department of Psychological Medicine, Centre for Affective Disorders*

*^2^ Department of Neuroimaging*

*^4^ Department of Biostatistics*

*Institute of Psychiatry, Psychology & Neuroscience, King's College London, United Kingdom*

*^3^ Cognitive and Behavioral Neuroscience Unit and Neuroinformatics Workgroup, D’Or Institute for Research and Education (IDOR) – Rio de Janeiro, Brazil*

*^5^ South London and Maudsley NHS Foundation Trust, Bethlem Royal Hospital, Monks Orchard Road, Beckenham, Kent, BR3 3BX, United Kingdom*

****Corresponding author***

*Dr Roland Zahn*

*Centre for Affective Disorders*

*Institute of Psychiatry, Psychology and Neuroscience (IoPPN), King's College London*

*Main Building, PO72*

*16 De Crespigny Park*

*London SE5 8AF*

*e-mail:* [*roland.zahn@kcl.ac.uk*](mailto:roland.zahn@kcl.ac.uk)

**Supplementary Methods**

***Exclusion criteria***

We excluded participants with significant risk of suicidality, violence or current self-harming behaviour. Additional exclusion criteria were defined as follows: Standard MRI contraindications, i.e. non-removable ferromagnetic devices or implants due to the possible dangerous effects of the MRI magnet upon metal objects in the body, a history of manic or hypomanic episodes, of schizophreniform symptoms or schizophrenia, or substance abuse, a history of neurological disorders such as seizures, loss of consciousness following brain injury or medical disorders affecting brain function, blood flow or metabolism, learning disabilities, major medical, developmental or relevant other axis-I disorders, prior specialist diagnosis of attention deficit hyperactivity disorder (ADHD), antisocial or borderline personality disorder, significant impairment of psychosocial functioning before the last major depressive episode (MDE) indicating the possibility of a comorbid personality disorder, current intake of benzodiazepines, GABAergic or benzodiazepine receptor agonists, current recreational drug use, past violence or current aggressive impulses, impairments of vision or hearing which cannot be corrected during the treatment sessions, or pregnancy.

***Pre-registered outcome measures***

The primary outcome measure was the BDI-II (Beck et al., 1996). Pre-registered secondary outcome measures were:

1. Montgomery-Åsberg Depression Rating Scale (MADRS, (Montgomery & Åsberg, 1979))
2. Quick Inventory of Depressive Symptomatology (QUIDS-SR16, (Rush et al., 2003))
3. Profile of Mood States (POMS) Scale (McNair et al., 1971) depression-dejection subscale
4. Rosenberg Self-Esteem Scale (Rosenberg, 1965)
5. In the fMRI neurofeedback group: decrease in post vs pre-training rSATL–posterior SC correlation for self-blame relative to blaming others between the first and last treatment session (i.e. at the start of visit 2 and at the end of visit 4), using fMRI as measured by regression coefficients for the time series, as extracted by the software FRIEND (Functional Real-time Interactive Endogeneous Neuromodulation and Decoding (Basilio et al., 2015; Sato et al., 2013))
6. Reduction in implicit self-blaming bias between pre-treatment visit 1 and post-treatment visit 5 (7-13 days after final treatment session) as assessed with the Brief Implicit Association Test (BIAT, Sriram & Greenwald, 2009, subcategories contempt–anger and contempt–anxiety, see supplementary methods)
7. Reduction in agency-incongruent self-blame between pre-treatment visit 1 and post-treatment visit 5 (7-13 days after final treatment session) as assessed with the short version of the value-related moral sentiment task (VMST, see supplementary methods (Zahn et al., 2015)).
8. Self- and observer-rated clinical global impression at post-treatment visit 5 (7-13 days after final treatment session) as assessed with the CGI-Scale (Busner et al., 2009).
9. Withdrawal rates
10. Adverse events
11. Self-rated self-blame as assessed with the mean of self-blame ratings of two self-blame-specific autobiographical events obtained at the first and last treatment session.
12. Observer-rated self-blame as assessed with the Moral Emotion Addendum to the AMDP depression (Faehndrich & Stieglitz, 1997, 2007; Zahn et al., 2015b) as the sum of all self-blaming emotion scores.

***Trial design modification***

Initially the NeuroMooD study was designed to compare three treatment arms, investigating the treatment effects of fMRI neurofeedback with an active, cathodal and a sham transcranial direct current stimulation (tDCS) intervention. Specifically, the original single-blind, randomised controlled design compared three sessions of the fMRI neurofeedback treatment as described above with three sessions of right superior temporal lobe cathodal tDCS plus self-guided psychological intervention and three sessions of sham right superior temporal lobe tDCS plus self-guided psychological intervention. Due to funding constraints, the original trial design had to be modified, however, and the data of the 6 randomised participants that had already been collected were discarded.

***Further randomisation details***

For stratified randomisation we categorised baseline BDI-II scores as follows: BDI-II scores below 14 points indicating minimal depression, BDI-II scores between 14 and 28 points comprising mild and moderate depression and BDI-II scores of 28 points or higher, implying severe depressive symptoms. Participants were informed about their allocated treatment group upon completion of the baseline clinical and neuropsychological testing on their pre-treatment assessment (visit 1).

***Further intervention details***

At the end of each treatment session, the participant’s suicide risk was assessed using the MINI International Neuropsychiatric Interview suicidality module, focussed on the time period since the previous study appointment. In addition, the severity of depressive symptoms was monitored and assessed with the BDI-II. Participants were excluded if they showed a suicide risk greater than low on the MINI and as judged by R.Z., or if their depressive symptoms had worsened, as reflected by an increase of 10 points or more on the BDI-II compared to the baseline score prior to randomisation. In such an instance, the protocol required un-blinding of the lead psychiatrist of the NeuroMooD trial (R.Z.), who discussed treatment recommendations with the patient if requested. By doing so, participants were assisted in accessing standard treatment options swiftly.

***NeuroMooD protocol violations***

Minor violations of the NeuroMooD protocol occurred during the course of the trial due to difficulties in scheduling participants’ treatment and final assessment visits. Modified schedules had to be arranged for individual participants who were unable to attend study visits within the preferred interval of 7 to 13 days between appointments because of other commitments. Also, limited availability of fMRI scanning slots at the MRI facilities of the Centre for Neuroimaging Sciences, King’s College London, affected MDD patients allocated to the fMRI neurofeedback group, occasionally causing a delay in the scheduling of treatment visits. As this issue became apparent early in the study, treatment visits for the psychological treatment group were scheduled in intervals comparable to those of the fMRI neurofeedback group. Ultimately, no significant differences were found between treatment groups regarding the total number of days included in the study (t=1.21, df=33, p=.237, two-tailed), considering the period from randomisation (visit 1) until trial completion (visit 5). On average, it took fMRI neurofeedback participants 40 days (SD=9.18) to complete the NeuroMooD trial. Similarly, participants randomised to the psychological intervention group participated on average for 37 days (SD=7.37).

***Assessment and evaluation of participants: clinical assessment***

The diagnostic, clinical and cognitive assessment comprised standardised, validated measures that have been used extensively in psychiatric research. We used the following standard clinical and cognitive instruments: Structured Clinical Interview (SCID) for DSM-5 (First et al., 2002); AMDP Psychopathology Interview questions on depression (Faehndrich & Stieglitz, 2007; Zahn et al., 2015b); Longitudinal Interval Follow-Up Evaluation (LIFE; (Keller et al., 1987)); Clinical Global Impression (CGI) Scale (Busner et al., 2009); Beck Depressive Inventory (BDI-II; (Beck et al., 1996)); Montgomery-Åsberg Depression Rating Scale (MADRS; (Montgomery & Åsberg, 1979)); Quick Inventory of Depressive Symptomatology (QUIDS-SR16; (Rush et al., 2003)); Hypomania Checklist-16 (Forty et al., 2010); Rosenberg Self-Esteem Scale (Rosenberg, 1965); Profile of Mood States (POMS) Scale (McNair et al., 1971); MINI International Neuropsychiatric Interview (module on suicidality only (Sheehan et al., 1998)); Psychiatric Family History Screen (Weissman et al., 2000); Life Events Questionnaire (Brugha et al., 1985); Childhood Trauma Questionnaire (CTQ; (Bernstein et al., 2003)); Altman Self-Rating Mania Scale (Altman et al., 1997 1997); Addenbrooke’s Cognitive Examination (ACE-III)(Hsieh et al., 2013) in patients >50 years only.

In addition to the abovementioned scales and measures, clinical evaluation of participants further entailed a non-structured clinical interview, as well as the documentation of the patient’s medical history and in women the day in their menstrual cycle. Furthermore, age at onset, episode duration(s), and total illness duration was recorded, along with details about the course of illness, i.e. number of episodes and medication history.

***Further psychological intervention details***

The intervention consisted of four parts. In the first and the fourth part, participants were asked to only think about the autobiographical events triggered by their cue words, without using any strategies to manage their feelings of self-blame. Before the second and third part of the intervention, participants were instructed to start using one or more self-guided strategies when seeing their self-blame cue words to manage their feelings of self-blame constructively.

Participants were given the following instructions before the treatment session was started:

’At the beginning and end of the session, you will have to think about the self-blame and anger events when shown your cue words. In between, you will be asked to keep thinking about the self-blame event while trying to use one or more strategies that best help you to cope with the self-blaming feeling. When numbers are presented on the screen, you will have to subtract seven from the number displayed.’

In the first and fourth part of the presentation, participants were shown their self-blame, and their other-blame cue words, parts two and three only contained the patient’s self-blame cue words and no other-blame-provoking cue words. Parts 1 and 4 were 408 seconds in length, including emotional blocks and mental subtraction blocks, plus a 30-second reminder of task instructions. Parts 2 and 3 consisted of a time sequence of 424 seconds each, containing self-blame cue words and subtraction blocks, in addition to the display of instruction slides for 60 seconds. Consequently, the intervention part of each treatment session was completed after approximately 30 minutes.

The order of the displayed cue words and numbers was as follows:

Part 1 of the intervention: instruction to only think about the autobiographical events without using psychological strategies 🡪 number 🡪 self-blame cue word 1 🡪 number 🡪 other-blame cue word 1 🡪 number 🡪 self-blame cue word 1 🡪 number 🡪 other-blame cue word 1 🡪 number 🡪 self-blame cue word 2 🡪 number 🡪 other-blame cue word 2 🡪 number 🡪 self-blame cue word 2 🡪 number 🡪 other-blame cue word 2.

Part 2 of the intervention: instruction to keep thinking about the events, while trying to use psychological strategies to cope with self-blaming feeling 🡪 number 🡪 self-blame cue word 1 🡪 number 🡪 self-blame cue word 1 🡪 number 🡪 self-blame cue word 2 🡪 number 🡪 self-blame cue word 2.

Part 3 was equal to part 2 of the intervention. Part 4 of the intervention was identical to Part 1. The mental subtraction blocks served as a distraction from the emotional load of the participants’ thought processes and to separate each emotional block. Self-blame cue words were presented in blue colour on a black background; other-blame cue words appeared in red on black background and numbers were presented in yellow.

In both treatment groups, participants were instructed to implement the psychological strategies in their everyday lives and to use them in-between treatment visits whenever feelings of self-blame would arise. The frequency of use was recorded at the next treatment visit. Participants were instructed to continue using the strategies until the final assessment visit (visit 5).

***Image acquisition details***

Image acquisition was carried out at the King’s College London Centre for Neuroimaging Sciences on an MR750 3.0T MR system (GE Healthcare, Chicago, USA), using a hyperbolic secant excitation pulse, optimised for orbitofrontal and inferior temporal regions, minimising signal dropout (Wastling & Barker, 2015). A 32-channel head coil was chosen to provide an optimal signal-to-noise ratio. Functional image acquisition was performed parallel to the AC-PC plane, top to bottom, using a T2*-weighted echo-planar imagining EPI (BOLD) sequence (TR = 2000 ms, TE = 30 ms, matrix = 64x64, FOV = 211 mm, flip angle = 73°, voxel size = 3x3x3 mm; slice thickness =3 mm, slice gap = 0.3 mm, 36 slices). An auto shimming procedure was applied. Four additional volumes, which were automatically discarded, were acquired at the start of each run to account for signal equilibration effects. High-resolution anatomical images were acquired with an inversion recovery–prepared spolied gradient echo (IR-SPGR) sequence (TR = 7.3 sec, TE = 3.0 sec, matrix = 256 x 256, FOV = 270 mm, slice thickness = 1.2 mm, 196 slices).

Clinical images were acquired on the first day of treatment (visit 2) using a T2-weighted fast spin echo sequence (2 mm thickness, 72 slices) and a FLAIR sequence (4 mm thickness, 36 slices) and checked for anatomical brain abnormalities after the treatment session by a radiologist at the scanning site, independent of additional, internal checks completed by the NeuroMooD study team.

While being in the MRI scanner, the participant’s head motion was restricted using padding and heart rate measurements recorded via a finger pulse sensor. Stimuli were projected onto a screen located behind the participant’s head, and a mirror fitted to the head coil allowed MDD patients to view the visual stimuli presented during image acquisition, i.e. autobiographical cue words and the visual feedback. Verbal instructions were communicated via the MRI intercom, participants, however, were instructed to respond using a button box placed in their hands to avoid incidental head movement.

***Additional experimental neuropsychological tasks***

Before and after each treatment session, participants rated the intensity of evoked self-blame and other-blame feelings on a Likert-type scale from 0 to 10. Moreover, they rated (from 0 to 10) how successful they felt in the emotional training during the intervention and estimated the percentage of time (0-100%) that they were able to focus during the session.

Additional experimental tasks developed by our research group were administered, designed to explore neurocognitive aspects of implicit self-contempt biases and self- and other-blaming emotions:

1. A modified short version of the value-related moral sentiment task (VMST; Zahn et al., 2015a): this computerised task investigates emotions related to self-blame (i.e. guilt, shame, self-contempt, self-disgust, self-directed anger) versus blaming others (indignation, anger, contempt or disgust towards others). Preceded by the description of hypothetical scenarios of social behaviours of the participants themselves and their best friends, participants are instructed to select the emotion they are most likely to experience. We added items related to action tendencies (Roseman et al., 1994), previously validated in an unpublished study. The following action tendencies were measured: creating distance from self, hiding, apologising, creating distance from friend, verbally or physically attacking/punishing friend or no action/other action. For this trial report we only focus on the pre-registered secondary outcome measure of agency-incongruent self-blaming emotions, so the percentage of other-agency trials where shame, guilt, or self-disgust/contempt were experienced. This was chosen because unpublished analyses of previous data showed a correlation of rsATL-posterior SC connectivity for self-blame vs. other-blame and agency-incongruent self-blaming emotions on the full version of the VMST (Lythe et al., 2015).
2. Brief Implicit Association Test (BIAT)(Sriram & Greenwald, 2009): this computerised task using Inquisit Software (www.millisecond.com) was developed by our research group by RZ and Dr Karen Lythe in collaboration with Profs Rüsch and Bodenhausen. It is an indirect measure of self-contempt bias, evaluating the association of contempt or disgust with oneself relative to others. The task design is based on similar tests that have been validated to measure implicit self-esteem (Greenwald & Farnham, 2000).
3. A modified version of the social knowledge differentiation task (Green et al., 2013): this computerised, neuropsychological test examines the participant’s ability to access differentiated social conceptual knowledge when instructed to appraise hypothetical scenarios of social behaviour of different contexts of agency (self-agency vs other-agency). The task was modified by restricting the original task to 30 items, focussing on negatively valenced scenarios only. This was not a pre-registered secondary outcome measure and the results will be reported in a separate paper.
4. Social agency inference task : Specifically developed for this research project, this computerised task assesses whether changes in the perception of social agency underpin self-blaming biases in MDD. This task was not a pre-registered secondary outcome measure and will be reported in a separate paper.

***Brief Implicit Association Test Design and Analysis***

The Brief Implicit Association Test (BIAT) measures implicit associations between two categories compared with another pair using two blocks of 20 stimuli each (16 used for the analysis). Participants were asked to press a left or right button depending on their categorisation of a word appearing on the screen. The Contempt vs. Anxiety BIAT consisted of four categories: Self-agency, Other-agency, Contempt, [Anxiety] as non-focal category. In Block 1, participants pressed the left key for “Participant acts” OR “Contempt”. They pressed the right key for everything else. In Block 2, they had to press the left key for “Friend acts” OR “Contempt” and pressed the right key for everything else. Stimuli presented for categorisation were: ”Participant acts”, “Participant does”, “Participant makes”, “Participant causes” as examples of Self-agency. The same stimuli, but with the best friend’s name were used for other-agency. “Hate”, “disgust”, “contemptuous”, “contempt” were used as examples of the Contempt category. “Anxiety”, “anxious”, “fear”, “scared” were used as examples of the Anxiety category. The Contempt vs. Anger BIAT was constructed in the same way, only replacing Anxiety with the Anger category and using “anger”, “angry”, “fury”, “furious” as examples for categorisation.

We computed D scores using the optimised scoring algorithm (Nosek, 2005) as response time means for Block 2 – means Block 1 / standard deviation across blocks. This means that participants with stronger biases towards associating contempt with self were expected to show faster responses in Block 1 and thus more positive BIAT D scores. Unlike other BIATs, we used self-agency and other-agency rather than just self and other. Due to an undetected technical error in the set-up of the BIAT, we used “Participant” and “Friend” instead of the first names of participants and friends which may limit the validity of the results.

The self-contempt BIATs were employed as indirect measures of self-contempt biases, evaluating the association of contempt or disgust with oneself relative to others. Further, we included an established BIAT (Sriram & Greenwald, 2009) used to assess implicit self-esteem (Greenwald et al., 1998), which has been validated to measure implicit self-esteem (Greenwald & Farnham, 2000). The reason for employing BIATs was to measure biases without the participants’ awareness of what the task captures. This strategy is meant to prevent distortions in the participants’ response due to social desirability.

BIAT data was exported from Inquisit 3 (https://www.millisecond.com/products/Inquisit3/) and analysed with SPSS 24 (<https://www.ibm.com/analytics/spss-statistics-software>). Scoring algorithms and analyses strategies were based on the improved scoring algorithm (Nosek, 2005): Trials with an error rate of higher 30% of 32 trials were excluded from the analyses; similarly, trials, where more than 10% of participant responses had a latency of less than 300 milliseconds, were excluded. Self-contempt bias was measured by subtracting the mean value of latency for the category ‘self and contempt’ (16 trials) minus the mean value of the latency for the category ‘other and contempt’ (16 trials), divided by the standard deviation of latency computed for all 32 trials. Hereby, a more positive total score is understood as being indicative of a higher degree of self-contempt.

**Supplementary Results**

***Adverse events and withdrawal rates***

Overall, six adverse events were reported following randomisation, three occurring after the first treatment session. A possible relationship with the study was suspected in four of the overall six adverse events throughout the trial, while no relationship was observed in one case and a probable relationship was assumed in one other instance. In the latter case, the participant reported transient insomnia lasting one night after his first fMRI neurofeedback session.

All adverse events were mild and constituted of two withdrawals and one incident of exclusion from the study (see details in Supplementary Table 7). One participant had to be excluded on the day of the first treatment session, after having been randomised to the psychological intervention group, as the patient presented with symptoms of depression that had worsened by 10 points on the BDI-II between baseline assessment and first intervention day. In addition to the six post-randomisation events mentioned above, one adverse event occurred prior to randomisation and had no relation with the study. The participant’s result on the ACE-III was suggestive of a neurological condition, and further referral to a cognitive assessment service was recommended.

Throughout the trial, seven participants withdrew their consent and ended their participation in the study, four prior to the first day of intervention and three at different time points following their first treatment session. Those participants withdrawing before the first treatment session reported to not feel well enough to participate or to experience time-related challenges that would make it impossible to attend further study appointments. The participants who withdrew after their first treatment session, described family or financial reasons for their decision. As mentioned above, transient insomnia in the night following the first fMRI neurofeedback session was cause for one MDD patient to discontinue trial participation.

**Supplementary Table 1|** Trial schedule chart

|  | Initial Patient Contact | Pre-Trial  Assessment  Visit 1 | Treatment Session  Visit 2 | Treatment Session  Visit 3 | Treatment Session  Visit 4 | Post-Trial Assessment  Visit 5 |
| --- | --- | --- | --- | --- | --- | --- |
|  | [email & phone] | [day 0] | [1-13 days  after visit 1] | [7-13 days  after visit 2] | [7-13 days  after visit 3] | [7-13 days  after visit 4] |
| Oral informed consent |  |  |  |  |  |  |
| Introduction to the clinical trial |  |  |  |  |  |  |
| Assessment of eligibility |  |  |  |  |  |  |
| Written informed consent |  |  |  |  |  |  |
| Clinical & neuro-  psychological assessment |  |  |  |  |  |  |
| Psychological Intervention only |  |  |  |  |  |  |
| fMRI neurofeedback training |  |  |  |  |  |  |
| Mood assessment |  |  |  |  |  |  |
| Risk assessment |  |  |  |  |  |  |

**Supplementary Table 2**| Clinical characteristics of intervention groups

|  | **PSYCHOLOGICAL (n=16)** | **NEUROFEEDBACK (n=19)** |
| --- | --- | --- |
| Number of previous MDEs (percentiles) | 25^th^=2 50^th^=4.5, 75^th^=13.75 | 25^th^=3 50^th^=4,  75^th^=8 |
|  | range: 2-66 | range: 1-110 |
| Current MDE  Partially remitted  **MDD DSM-5 subtype**  Anxious Distress  Melancholic Features  Melancholic Features + Anxious Distress  Atypical Features  Atypical Features + Anxious Distress  None | 9  7  4  1  3  0  1  7 | 10  9  11  2  2  1  0  3 |
| **Current medication** |  |  |
| Psychotropic medication  Antidepressant (therapeutic dose)  Of which SSRI | 10  9  6 | 10  9  6 |
| **Life-time co-morbidity** |  |  |
| Current Persistent Depressive Disorder of the dysthymic subtype  Past PTSD with residual symptoms  Past PTSD fully remitted  Current Social Anxiety Disorder  Past Social Anxiety Disorder | 2  2  0  1  0 | 3  3  1  2  2 |
| Past Anorexia Nervosa | 0 | 1 |

Participants in the psychological intervention and fMRI neurofeedback groups did not differ on median numbers of previous episodes despite higher percentiles in the psychological intervention group (U=145, p=.832, two-tailed). MDE = major depressive episode, MDD = major depressive disorder, SSRI = selective serotonin reuptake inhibitor, SNRI = serotonin-norepinephrine reuptake inhibitor, PTSD = posttraumatic stress disorder; M = mean, SD = standard deviation, [M-M] = Minimum-Maximum. In the fMRI neurofeedback group, one participant was suspected of displaying symptoms of autism spectrum disorder; one participant showed symptoms of attention deficit hyperactivity disorder during childhood, one participant reported past heavy alcohol and substance use.

**Supplementary Table 3|** Demographic characteristics of intervention groups

|  | **PSYCHOLOGICAL** | **NEUROFEEDBACK** |
| --- | --- | --- |
|  | **M;SD;[Min-Max]** | **M;SD;[Min-Max]** |
| Age in years  Years of education | 37.63;9.74;[22-55]  18.06;2.52;[13-22] | 36,74;11.04;[20-59]  16.95;3.15;[11-23] |

Participants of both treatment groups did not differ in age (t=-2.50, df=33, p=.804) or years of education (t=-1.14, df=33, p=.262). In the psychological intervention group, 17/22 were female (77%) and 17/21 in the neurofeedback group (81%, Contingency Coefficient=.05, p=.77, n=43).

**Supplementary Table 4**| Comparison of anxious and non-anxious distress subgroups

| Measure | **Anxious MDD** | **Non-anxious**  **MDD** | **Statistic** | **Significance** |
| --- | --- | --- | --- | --- |
| Years of education | 17.3±2.6 | 17.3±3.4 | t=0.02 | .98 |
| Age | 38.6±10.2 | 34.2±8.4 | t=-1.4 | .16 |
| Gender | 22/28 female (79%) | 12/15 female (80%) | CC=.02 | .91 |
| Number of previous MDEs (median) | 3.5 | 5 | Median Test ꭓ2=.11 | .74 |
| Current antidepressant medication at therapeutic dose (n) | 15/28 (53.6%) | 7/15 (47.7%) | CC=.07 | .67 |
| BDI-II at Baseline | 29.9±9.6 | 27.8±6.8 | t=-.7 | .46 |
| Frequency of therapeutic  strategy use (m) | 9.1±2.5 | 7.6±3.1 | t=-1.4 | .17 |
| AMDP Anxiety (n) | 19/28 (67.9%) | 5/15 (33.3%) | CC=.32 | .03 |
| AMDP Anger towards others (n) | 10/28 (35.7%) | 0/15 (0.0%) | CC=.37 | .008 |
| Sum of self-blaming emotions score (median) | 2.5 | 2 | Median Test ꭓ2=.09 | .76 |
| Any self-blaming emotion (n) | 25/28 (89.3%) | 10/15 (66.6%) | CC=.27 | .07 |

Anxious distress features were defined on the Structured Clinical Interview for DSM5 for the current episode. MDE = major depressive episode, MDD = major depressive disorder, BDI-II = Beck Depression Inventory-II; m = mean, ± = standard deviation, [M-M] = Minimum-Maximum. CC=Contingency Coefficient in Crosstab statistics. The frequency of therapeutic strategy was assessed at visit 5 and was collected in all n=35 completers (n=21 anxious MDD, n=14 non-anxious MDD). All other measures were collected at baseline in all randomised patients (n=43, n=28 with and n=15 without anxious distress). Baseline psychopathological measures (AMDP anxiety and anger towards others) were based on binarising scores for the past two weeks into 0=absent or mild or 1=moderate or severe as previously (Zahn et al., 2015b). The sum of self-blaming emotions score on the AMDP was based on our secondary outcome measure as described above. The any self-blaming emotion measure was based on identifying patients who had at least one self-blaming emotion to a moderate degree on the moral emotion addendum to the AMDP (i.e self-directed anger, shame, guilt, or self-disgust/contempt). Significance reflects 2-sided p-values or approximate significance for Contigency Coefficient statistics. There were only 8 of 43 patients (19%) who did not experience at least one self-blaming emotion to a bothering degree on the AMDP, but this absence of self-blaming emotions was not associated with anxious distress, on the contrary, there was a trend towards anxious distress patients showing more consistent self-blaming emotions on our interview. This indicates that both self- and other-blaming emotions were prominent in the anxious distress subtype.

**Supplementary Table 5|** Secondary correlation analyses

**Correlation is significant at the 0.01 level (2-tailed). *Correlation is significant at the 0.05 level (2-tailed). Difference scores were computed by subtracting post- vs pre-treatment scores.

**Supplementary Table 6|** Means and standard deviations for pre-registered non-continuous secondary outcome measures collected pre- and post-intervention

| MEASURE [sample size] | **PRE-**  **INTERVENTION** | | | | **POST-**  **INTERVENTION** | | | | |
| --- | --- | --- | --- | --- | --- | --- | --- | --- | --- |
|  | **PSYCH** | | **NFB** | | **PSYCH** | | **NFB** | | |
|  | m | sd | m | sd | m | sd | | m | sd |
| Participant-Rated Self-Blame  [PRE:39; POST:35] | 8.69 | 0.94 | 8.07 | 1.49 | 5.56 | 2.39 | | 4.16 | 2.19 |
| Observer-Rated Self-Blame  [PRE:43; POST:35] | 3.05 | 2.01 | 2.82 | 1.82 | 1.75 | 1.18 | | 1.53 | 1.31 |

Participant-rated self-blame scores are based on the mean of two autobiographical events per subject; Observer-rated self-blame was defined as per trial register as the sum of all self-blaming emotion scores (guilt/shame, self-directed anger, and self-disgust/contempt/hate/loathing, each of these 3 emotion scores is scored on a scale from 0=absent, 1=mild/minimal, 2=moderate, 3=overgeneralised and severe, resulting in a minimum sum score of 0 and maximum sum score of 9) are based on the moral emotion addendum of the AMDP Psychopathology Interview questions on depression(Faehndrich & Stieglitz, 2007; Zahn et al., 2015)). PSYCH = psychological intervention group, NFB = fMRI neurofeedback group. m=mean, sd=standard deviation, df = degrees of freedom. For medians and range, as well as non-parametric statistics for the self-blame measures, please see Table 3 in main manuscript.

**Supplementary Table 7|** Detailed description of adverse events

|  | **Time of occurrence** | **Treatment group** | **Severity of event** | **Specifics/Summary of event** | **Relation-ship to study** | **Action taken/ Consequences** | **Further Actions** |
| --- | --- | --- | --- | --- | --- | --- | --- |
| 1 | Incident occurred at baseline assessment visit (Visit 1). | n/a  *[Participant was not randomised.]* | mild | Participant had a below normal total score on the ACE-III (82/100) indicating a mild cognitive impairment.  Participant withdrew consent for report to be sent to them and their GP, although advised to repeat testing once the depressive symptoms have improved in order exclude a relevant cognitive impairment which was not due to depression, although pattern of impairment was not typical of early Alzheimer’s disease. Participant was fully independent in daily activities with no clinical indication of dementia. Neurological exam detected no abnormalities (cranial nerves intact, no signs of limb weakness or rigidity, normal gait, no tremor). | none | Participant was not randomised and excluded from study permanently. | We offered to be contacted for further advice.  PI was notified. |
| 2 | Incident occurred between the first and the second study visit, hence prior to the first treatment visit (Visit 2). | fMRI neurofeedback | mild | Participant reported to have hit themselves  Participant explained that they felt very bad about themselves over the weekend which was stressful. This caused the participant to feel very low. In addition to that choosing the self-blaming and indignation cue words for the study was on the participant’s mind. According to the participant all this combined made them feel low which led to the described self-harming behaviour. Participant did not cause themselves any injuries. | possible | Risk assessment:  Thoughts of being better off dead, thoughts of hurting themselves and deliberate self-harming behaviour since Visit 1.  Participant was asked if they would like to continue with the study, if they thought that they would benefit from it at all and if they would like to speak with the PI to discuss this further. Participant responded that they would like to continue with the study and that there would be no need to speak with PI.  Participant continued in the study. | PI was notified. |
| 3 | Incident occurred between the first and the second study visit, hence prior to the first treatment visit (Visit 2). | Psychological Intervention | mild | Participant reported suicidal thoughts which they found difficult to control before the first treatment session after thinking about their autobiographical self-blaming situation. Nevertheless, the participant was able to control their thoughts and reported to never seriously consider acting on them. | possible | Risk assessment:  MINI suicidality screen: 9 Points, moderate on the day.  PI called participant for a phone consultation. Participant reported to feel much better after the treatment session on the previous day and that the psychological strategies, the researcher introduced him to, helped. The participant reported that their feelings of self-blame reduced as well as their suicidal thoughts. Participant had no suicidal thoughts on the day, showed good rapport, no aggression or hypomanic signs and was very credibly, able to promise getting in touch with us should participant feel worse.  Participant continued in the study. | Participant was informed that they can request a call back also over the weekend from PI.  Following the participant’s consent, a letter was sent to the participant’s GP, copied to participant, to keep GP informed.  PI was notified. |
| 4 | Incident occurred between the first and the second study visit, hence prior to the first treatment visit (Visit 2). | Psychological Intervention | mild | BDI worsened 10 points between assessment visit (Visit 1) and first treatment visit (Visit 2) which was related to life events.  Participant explained that they do not think the assessment visit or choosing the guilt/self-blame and indignation cue words for the study made them feel low, but combined with the current life situation it may have contributed to the increase in severity of symptoms. | possible | Risk assessment:  Thoughts of being better off dead and thoughts of wanting to hurt themselves, but able to control these impulses. No change in suicidality risk since Visit 1. Overall low suicide risk since Visit 1.  Participant was advised that per protocol they would not be able to continue with the study. Participant felt upset about this but understanding.  Participant was offered advice on other treatment options, letter was sent to GP, copied to participant.  Participant was excluded from study permanently. | We offered to be contacted for further advice.  PI was notified. |
| 5 | Incident occurred prior to study visit 4. | Psychological Intervention | mild | Participant withdrew from study (before Visit 4) and explained by email that they would not be able to afford further participation in the study.  Participant explained that this was due to a change in their financial situation which meant they could not afford to lost time participating in the study.  *[Previously, participant had mentioned that they need to take off time from work in order to attend study visits (is paid by hour) and pointed out that train tickets to attend study appointments would be expensive.]* | possible | Monitoring and risk assessment on last study visit (Visit 3): BDI had dropped from 24 points (Visit 1) to 15 points (Visit 3), no change in suicidality risk since Visit 1  Participant withdrew/ was excluded from study permanently. | PI was notified. |
| 6 | Before Visit 4 | Psychological Intervention | mild | Arguments with their partner triggered the participant to have suicidal thoughts and very low energy. Participant was unable to go to work one day because of feeling very low. Since this incident, suicidal thoughts occurred often and presented with severe intensity. Participant made an agreement with a friend not to act on suicidal impulses. Participant also promised not to act on their thoughts while being included in the study. Participant thinks they are in control of them.  Participant reported thoughts of wanting to harm and injure themselves and had thoughts about being better off dead. Started uncontrollably/involuntarily hitting her head with her flat hand for a couple of seconds during arguments with partner, not causing herself any injuries. | none  *[Participant reported that the study appointments are helpful.]* | Risk assessment: no suicide plans; BDI-II: 24 points on the day (Visit 4) compared to 26 points on Visit 1.  Participant continued  in the study. | PI was notified.  PI called participant for a phone consultation. Participant reported that suicidal thoughts are under control. Participants expressed that they want to continue with the study.  PI offered that participant can get in touch with him via email to request a‎ call back if necessary; participant also has a former therapist who has offered to be contacted on the phone should participant feel unwell. |
| 7 | After Visit 2 (first treatment session) | fMRI neurofeedback | mild | Participant reported having experienced ‘intrusive memories of being in the scanner’, insomnia and ‘unpleasant feelings in their gut’ in the night that followed their first MRI neurofeedback treatment session (Visit 2).  Despite feeling better after one day, the participant withdrew from the study 5 days later. | probable  *[On day of the neurofeedback treatment session, the participant had reported to feel nervous but also positively excited about the experience. Participant did not report any unpleasant feelings immediately after having completed the scanning session.]* | Risk assessment:  MINI suicidality screen: low (0 points since Visit 1); BDI-II: 20 points (Visit 2) compared to 26 points on Visit 1.  Participant withdrew/ was excluded from study permanently.  MINI | PI was notified.  The participant’s GP was notified about his withdrawal from the study. PI offered to see participant again to make further treatment suggestions if needed. |

**
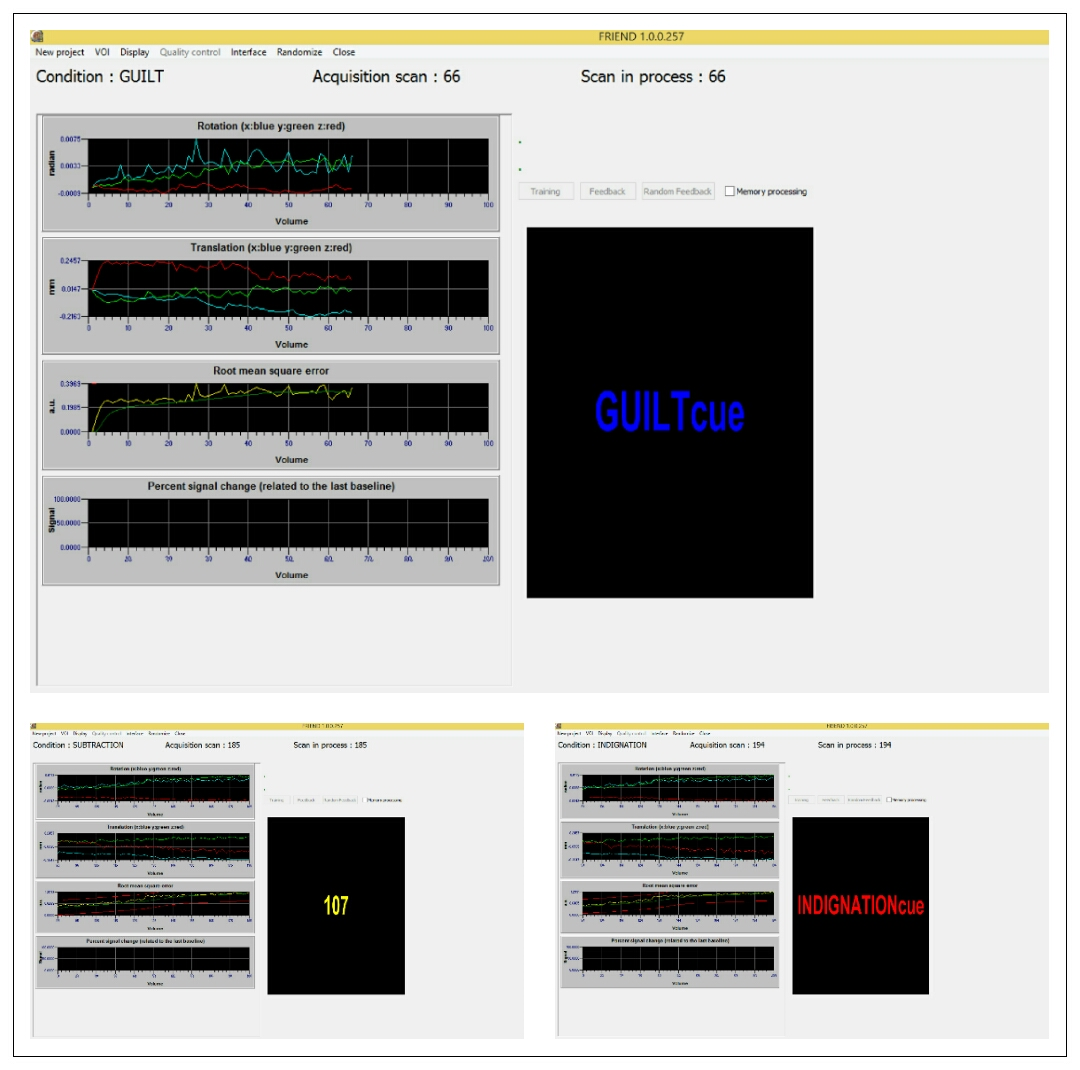
**

**Supplementary Figure 1**

Display of the interface of the fMRI neurofeedback software FRIEND during a data acquisition run (Basilio et al., 2015; Sato et al., 2013). During acquisition runs (run 1 and 4), participants were presented with their self-blame and other-blame/anger cue words. Cue words refer to autobiographical memories of events that trigger patients to experience feelings of self-blame or other-blame/anger. During these runs, participants were solely thinking about these events and are not using any psychological strategies to manage associated feelings of self-blame. In-between the emotional conditions, participants were presented with numbers which cue them to perform mental subtractions from a number displayed at the screen. The right panel displays what the participant sees in the scanner, the left panel displays motion parameters and percent signal change in the regions of interest.

**
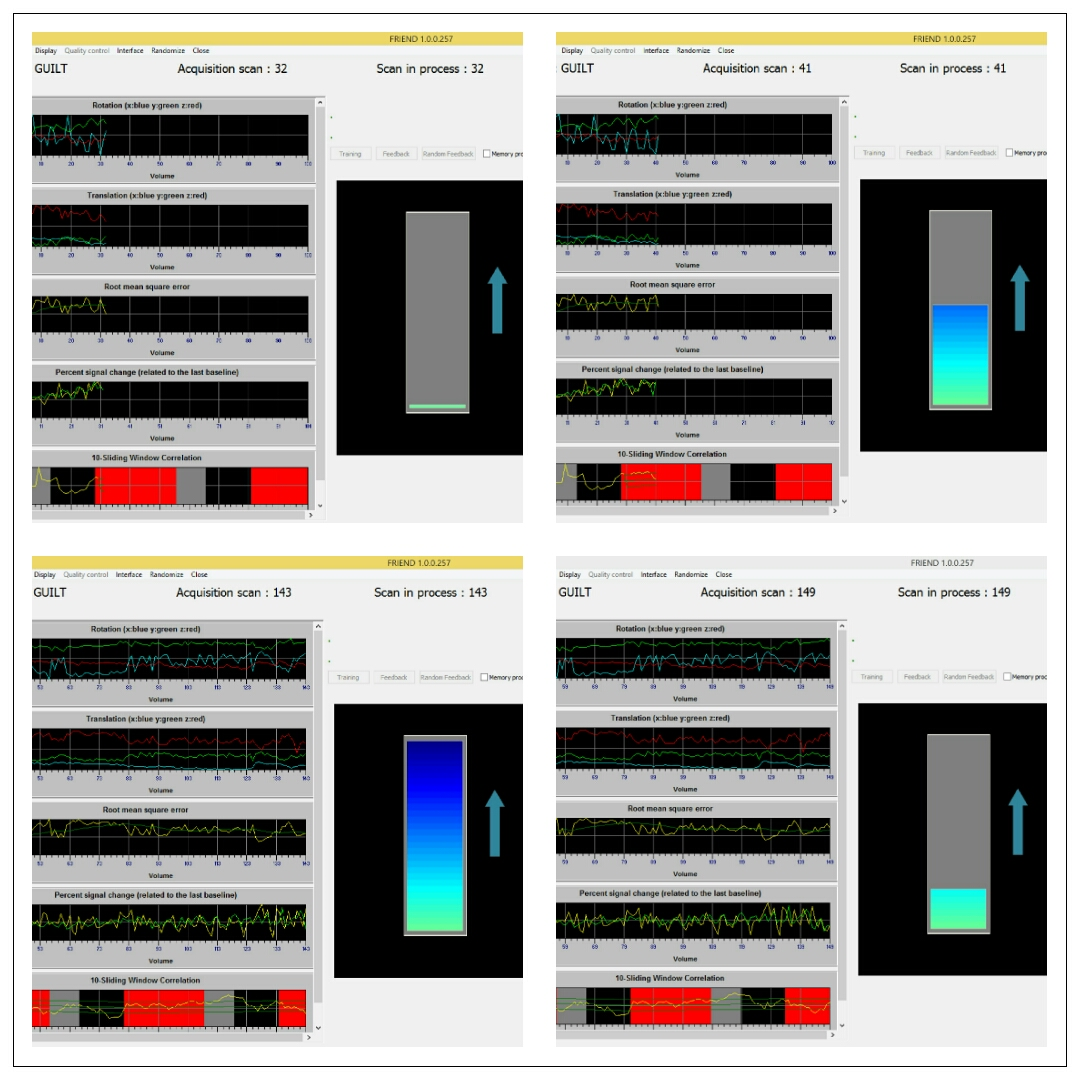
**

**Supplementary Figure 2**

Display of the interface of the fMRI neurofeedback software FRIEND during a neurofeedback training run. In the scanner, participants were presented with their individualised self-blame cue words or subtraction blocks. In the self-blame condition, a thermometer display containing a moving colour bar appeared and represented visual feedback of the patient’s functional connectivity patterns in real-time. During the fMRI neurofeedback runs (run 2 and 3), participants were asked to bring up the level of the thermometer by using psychological strategies when thinking about autobiographical self-blame-evoking events. The colour bar rises if functional (hyper-)connectivity between the rSATL and posterior SC successfully decreases.

**Supplementary Figure 3|** Consort Trial flow diagram

**E**

**N**

**R**

**O**

**L**

**M**

**E**

**N**

**T**

Phone screened for eligibility

(n=311)

Assessed for eligibility at visit 1

(n=71)

Excluded (n=28)

- - - Not meeting inclusion criteria (n=28)
    - Declined to participate (n=0)
    - Other reasons (n=0)

Randomised (n=43)

**ALLOCATION**

Allocated to psychological intervention (n=21)

- - - Received allocated intervention (n=18)
    - Did not receive allocated intervention (n=3; 2 withdrawals before visit 2 because of feeling too unwell to participate, 1 withdrawal before visit 2 due to time constraints)

Allocated to fMRI NF intervention (n=22); ‘Intervention’ refers to a minimum of 1of 3 allocated treatment sessions

- - - Received allocated intervention (n=21)
    - Did not receive allocated intervention (n=1; withdrew before visit 2 because of feeling too unwell to participate)

**FOLLOW-UP**

Lost to follow-up (n=0)

Discontinued intervention (n=2; 1 exclusion after visit 2 due to worsening of symptoms; 1 withdrawal after visit 3 due to financial reasons)

Lost to follow-up (n=0)

Discontinued intervention (n=2; 2 withdrawals after visit 2, 1 due to familial reasons, 1 due to occurrence of insomnia after fMRI NF session)

**ANALYSIS**

Analysed (n=16)

Excluded from analysis of primary outcome measure (n=0)

Analysed (n=19)

Excluded from analysis of primary outcome measure (n=0)

**Supplementary Figure 4|** Neurofeedback Success: Thermometer Scale Position

**Supplementary Figure 4|** Participants were successful in controlling the neurofeedback thermometer on average with positive feedback around the 50% mark of the thermometer scale (0-100% range). Participants’ neurofeedback success occurred already in the first session and was stable throughout further sessions with a slight drop in the final active run. FRIEND’s moving target algorithm means that after successfully reducing rSATL-posterior SC connectivity, it may be increasingly difficult to further successfully reduce it in subsequent training runs.

**Supplementary Figure 5|** Functional connectivity changes over training sessions


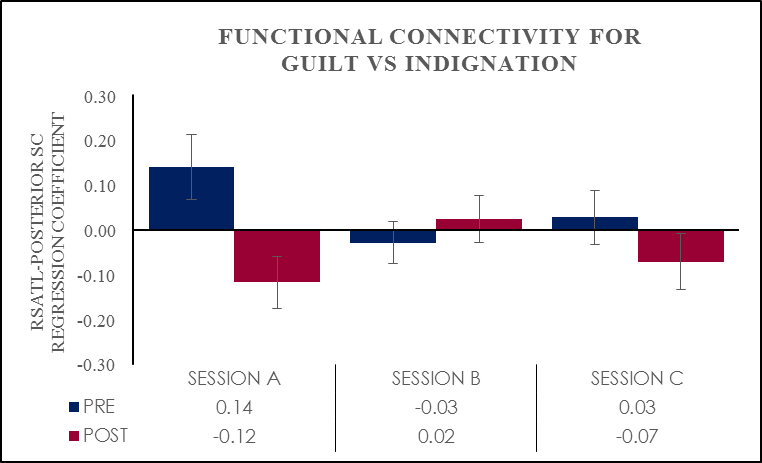


**Supplementary Figure 5|** Change in functional connectivity between rSATL and posterior SC in the self-blame vs other-blame condition, measured as Cohen’s D for regression coefficient means with standard errors for time series pre- and post-fMRI neurofeedback training in n=18 participants plotted for each neurofeedback session. A repeated measures ANOVA was conducted to investigate differences in rSATL-posterior SC functional connectivity pre- vs post-intervention in the self-blame vs other-blame condition over the course of all three treatment sessions. This analysis approach was chosen to contrast the two psychological conditions and thereby control for non-specific correlations, which make up a large fraction of the signal when considering each condition in isolation. While a significant main effect was found for pre- vs post-intervention (F(1,17)=4.5, p=.049, Wilks’ Lambda=.79, ηp²=.21), there was only a trendwise interaction between session and pre- vs post-interventional rSATL- posterior SC connectivity (F(2,16)=2.79, p=.091, Wilks’ Lambda=.74, ηp²=.26). As one can see in the plot above, self-blame connectivity was indeed reduced after the neurofeedback training relative to other-blame in concordance with our main analysis. It appears that most of this training effect occurred already after the first session (Session A), but this observation was only supported by a trendwise interaction between session and intervention effect (see above).

**Supplementary Reference**

Altman, E. G., Hedeker, D., Peterson, J. L., & Davis, J. M. (1997, Nov 15). The Altman Self-Rating Mania Scale. *Biological Psychiatry, 42*(10), 948-955. <https://doi.org/10.1016/S0006-3223(96)00548-3>

Basilio, R., Garrido, G. J., Sato, J. R., Hoefle, S., Melo, B. R., Pamplona, F. A., Zahn, R., & Moll, J. (2015). FRIEND Engine Framework: a real time neurofeedback client-server system for neuroimaging studies. *Frontiers in Behavioral Neuroscience, 9*, 3. <https://doi.org/10.3389/fnbeh.2015.00003>

Beck, A. T., Steer, R. A., & Brown, G. K. (1996). *Beck Depression Inventory-II*. Psychological Corporation.

Bernstein, D. P., Stein, J. A., Newcomb, M. D., Walker, E., Pogge, D., Ahluvalia, T., Stokes, J., Handelsman, L., Medrano, M., Desmond, D., & Zule, W. (2003, Feb). Development and validation of a brief screening version of the Childhood Trauma Questionnaire. *Child Abuse and Neglect, 27*(2), 169-190. <http://www.ncbi.nlm.nih.gov/pubmed/12615092>

Brugha, T., Bebbington, P., Tennant, C., & Hurry, J. (1985, Feb). The List of Threatening Experiences: a subset of 12 life event categories with considerable long-term contextual threat. *Psychol Med, 15*(1), 189-194. <http://www.ncbi.nlm.nih.gov/pubmed/3991833>

Busner, J., Targum, S. D., & Miller, D. S. (2009, May-Jun). The Clinical Global Impressions scale: errors in understanding and use. *Comprehensive Psychiatry, 50*(3), 257-262. <https://doi.org/10.1016/j.comppsych.2008.08.005>

Faehndrich, E., & Stieglitz, R. D. (1997). *Das AMDP-System, Manual zur Dokumentation psychiatrischer Befunde* (6th ed.). Hogrefe Verlag.

Faehndrich, E., & Stieglitz, R. D. (2007). *Leitfaden zur Erfassung des psychopathologischen Befundes, Halbstrukturiertes Interview anhand des AMDP-Systems* (3rd ed.). Hogrefe Verlag.

First, M. B., Spitzer, R. L., Gibbon, M., & Williams, J. B. W. (2002). *Structured Clinical Interview for DSM-IV-TR Axis I Disorders, Research Version, Patient Edition. (SCID-I/P)*. Biometrics Research, New York State Psychiatric Institute.

Forty, L., Kelly, M., Jones, L., Jones, I., Barnes, E., Caesar, S., Fraser, C., Gordon-Smith, K., Griffiths, E., Craddock, N., & Smith, D. J. (2010, 2010/08/01/). Reducing the Hypomania Checklist (HCL-32) to a 16-item version. *Journal of Affective Disorders, 124*(3), 351-356. <https://doi.org/https://doi.org/10.1016/j.jad.2010.01.004>

Green, S., Moll, J., Deakin, J. F., Hulleman, J., & Zahn, R. (2013). Proneness to decreased negative emotions in major depressive disorder when blaming others rather than oneself. *Psychopathology, 46*(1), 34-44. <https://doi.org/10.1159/000338632>

Greenwald, A. G., & Farnham, S. D. (2000, Dec). Using the implicit association test to measure self-esteem and self-concept. *Journal of Personality and Social Psychology, 79*(6), 1022-1038. <https://www.ncbi.nlm.nih.gov/pubmed/11138752>

Greenwald, A. G., McGhee, D. E., & Schwartz, J. L. (1998, Jun). Measuring individual differences in implicit cognition: the implicit association test. *Journal of Personality and Social Psychology, 74*(6), 1464-1480. <https://www.ncbi.nlm.nih.gov/pubmed/9654756>

Hsieh, S., Schubert, S., Hoon, C., Mioshi, E., & Hodges, J. R. (2013). Validation of the Addenbrooke's Cognitive Examination III in frontotemporal dementia and Alzheimer's disease. *Dementia and Geriatric Cognitive Disorders, 36*(3-4), 242-250. <https://doi.org/10.1159/000351671>

Keller, M. B., Lavori, P. W., Friedman, B., Nielsen, E., Endicott, J., McDonald-Scott, P., & Andreasen, N. C. (1987, Jun). The Longitudinal Interval Follow-up Evaluation. A comprehensive method for assessing outcome in prospective longitudinal studies. *Archives of General Psychiatry, 44*(6), 540-548. <https://www.ncbi.nlm.nih.gov/pubmed/3579500>

Lythe, K. E., Moll, J., Gethin, J. A., Workman, C. I., Green, S., Lambon Ralph, M. A., Deakin, J. F., & Zahn, R. (2015, Nov). Self-blame-Selective Hyperconnectivity Between Anterior Temporal and Subgenual Cortices and Prediction of Recurrent Depressive Episodes. *JAMA Psychiatry, 72*(11), 1119-1126. <https://doi.org/10.1001/jamapsychiatry.2015.1813>

McNair, D., Lorr, M., & Doppleman, L. (1971). *POMS Manual for the Profile of Mood States.* Educational and Industrial Testing Service.

Montgomery, S. A., & Åsberg, M. (1979). A new depression scale designed to be sensitive to change. *British Journal of Psychiatry, 134*(4), 382-389.

Nosek, B. A. (2005). *SAS MACRO FOR SCORING THE IMPLICIT ASSOCIATION TEST.* In <http://projectimplicit.net/nosek/papers/scoringalgorithm.sas.txt>

Roseman, I. J., Wiest, C., & Swartz, T. S. (1994). Phenomenology, behaviors, and goals differentiate discrete emotions. *Journal of Personality and Social Psychology, 67*(2), 206-221. <https://doi.org/10.1037/0022-3514.67.2.206>

Rosenberg, M. (1965). *Society and the Adolescent Self-Image*. Princeton University Press.

Rush, A. J., Trivedi, M. H., Ibrahim, H. M., Carmody, T. J., Arnow, B., Klein, D. N., Markowitz, J. C., Ninan, P. T., Kornstein, S., Manber, R., Thase, M. E., Kocsis, J. H., & Keller, M. B. (2003, Sep 01). The 16-Item Quick Inventory of Depressive Symptomatology (QIDS), clinician rating (QIDS-C), and self-report (QIDS-SR): a psychometric evaluation in patients with chronic major depression. *Biological Psychiatry, 54*(5), 573-583. <http://www.ncbi.nlm.nih.gov/pubmed/12946886>

Sato, J. R., Basilio, R., Paiva, F. F., Garrido, G. J., Bramati, I. E., Bado, P., Tovar-Moll, F., Zahn, R., & Moll, J. (2013). Real-time fMRI pattern decoding and neurofeedback using FRIEND: an FSL-integrated BCI toolbox. *PloS One, 8*(12), e81658. <https://doi.org/10.1371/journal.pone.0081658>

Sheehan, D. V., Lecrubier, Y., Sheehan, K. H., Amorim, P., Janavs, J., Weiller, E., Hergueta, T., Baker, R., & Dunbar, G. C. (1998). The Mini-International Neuropsychiatric Interview (M.I.N.I.): the development and validation of a structured diagnostic psychiatric interview for DSM-IV and ICD-10. *Journal of Clinical Psychiatry, 59 Suppl 20*, 22-33;quiz 34-57. <https://www.ncbi.nlm.nih.gov/pubmed/9881538>

Sriram, N., & Greenwald, A. G. (2009). The Brief Implicit Association Test. *Experimental Psychology, 56*(4), 283-294. <https://doi.org/10.1027/1618-3169.56.4.283>

Wastling, S. J., & Barker, G. J. (2015, Sep). Designing hyperbolic secant excitation pulses to reduce signal dropout in gradient-echo echo-planar imaging. *Magnetic Resonance in Medicine, 74*(3), 661-672. <https://doi.org/10.1002/mrm.25444>

Weissman, M. M., Wickramaratne, P., Adams, P., Wolk, S., Verdeli, H., & Olfson, M. (2000). Brief screening for family psychiatric history: The family history screen. *Archives of General Psychiatry, 57*(7), 675-682. <https://doi.org/10-1001/pubs.Arch> Gen Psychiatry-ISSN-0003-990x-57-7-yoa8214

Zahn, R., Lythe, K. E., Gethin, J. A., Green, S., Deakin, J. F., Workman, C., & Moll, J. (2015, Jun). Negative emotions towards others are diminished in remitted major depression. *European Psychiatry, 30*(4), 448-453. <https://doi.org/10.1016/j.eurpsy.2015.02.005>

Zahn, R., Lythe, K. E., Gethin, J. A., Green, S., Deakin, J. F., Young, A. H., & Moll, J. (2015b, Nov 1). The role of self-blame and worthlessness in the psychopathology of major depressive disorder. *Journal of Affective Disorders, 186*, 337-341. <https://doi.org/10.1016/j.jad.2015.08.001>
